# Supercritical CO_2_ Foaming of Radiation Cross-Linked Isotactic Polypropylene in the Presence of TAIC

**DOI:** 10.3390/molecules21121660

**Published:** 2016-12-07

**Authors:** Chen-Guang Yang, Mou-Hua Wang, Ming-Xing Zhang, Xiao-Hu Li, Hong-Long Wang, Zhe Xing, Lin-Feng Ye, Guo-Zhong Wu

**Affiliations:** 1 Shanghai Institute of Applied Physics, Chinese Academy of Sciences, Jialuo Road 2019, Jiading, Shanghai 201800, China; yangchenguang@sinap.ac.cn or yangchg@shanghaitech.edu.cn (C.-G.Y.); wangmouhua@sinap.ac.cn (M.-H.W.); zhangmingxing@sinap.ac.cn (M.-X.Z.); lixiaohushdx@163.com (X.-H.L.); wanghonglong@sinap.ac.cn (H.-L.W.); xingzhe@sinap.ac.cn (Z.X.); yelinfeng@sinap.ac.cn (L.-F.Y.); 2 University of China Academy of Sciences, Beijing 100049, China; 3 School of Physical Science and Technology, ShanghaiTech University, Haike Road 100, Pudong, Shanghai 201210, China

**Keywords:** polypropylene, TAIC, radiation cross-linking, supercritical carbon dioxide, foaming

## Abstract

Since the maximum foaming temperature window is only about 4 °C for supercritical CO_2_ (*sc*CO_2_) foaming of pristine polypropylene, it is important to raise the melt strength of polypropylene in order to more easily achieve *sc*CO_2_ foaming. In this work, radiation cross-linked isotactic polypropylene, assisted by the addition of a polyfunctional monomer (triallylisocyanurate, TAIC), was employed in the *sc*CO_2_ foaming process in order to understand the benefits of radiation cross-linking. Due to significantly enhanced melt strength and the decreased degree of crystallinity caused by cross-linking, the *sc*CO_2_ foaming behavior of polypropylene was dramatically changed. The cell size distribution, cell diameter, cell density, volume expansion ratio, and foaming rate of radiation-cross-linked polypropylene under different foaming conditions were analyzed and compared. It was found that radiation cross-linking favors the foamability and formation of well-defined cell structures. The optimal absorbed dose with the addition of 2 wt % TAIC was 30 kGy. Additionally, the foaming temperature window was expanded to about 8 °C, making the handling of *sc*CO_2_ foaming of isotactic polypropylene much easier.

## 1. Introduction

Polypropylene (PP) foam has been considered as a substitute for other thermoplastic foams, as its mechanical properties are enhanced; these include increased toughness, impact strength, stiffness-to-weight ratio, fatigue life, and thermal stability, as well as its relatively high service temperature [1,2,3,4,5,6]. PP foam has been used in electronic packaging, food packaging, construction materials, traffic equipment, sports equipment, and thermal and sound insulators. The application of PP foam is determined by its mechanical properties, which are dependent on the cell structure—such as cell type, cell size, cell size distribution, and cell density—as well as the foaming rate of PP [1,7,8,9]. Compared with traditional blowing agents, foaming using supercritical carbon dioxide (*sc*CO_2_) is particularly appealing, as *sc*CO_2_ can be easily dissolved in polymers, and the foaming process is environmentally friendly [9,10,11,12,13].

The low melt strength and narrow foaming temperature window are key constraints for the *sc*CO_2_ foaming of polypropylene [14]. Radiation cross-linking can be applied to improve the melt strength and thermal and mechanical properties of polypropylene, owing to the formation of a 3D network structure in the amorphous region [15,16,17]. However, compared to polyethylene, isotactic polypropylene more easily undergoes degradation upon irradiation, resulting in a poorer resistance to weathering [18,19]. Many studies have been performed on the foaming of PP by *sc*CO_2_ [20,21,22]. The melt strength and mechanical and heat-resistant properties of polypropylene have been shown to be significantly improved by irradiation in the presence of a cross-linking agent [23].

The main objective of this work is to investigate the *sc*CO_2_ foaming behavior of radiation cross-linked polypropylene with the addition of 2 wt % of triallylisocyanurate (TAIC), and PP with the addition of TAIC is defined as PP/TAIC. Radiation effects of the cross-linking agent on polypropylene, in terms of crystallinity, glass transition temperature, and foaming morphology, are also reported. Moreover, the impact of radiation cross-linking on cell diameter, cell density, foaming rate, and volume expansion ratio of polypropylene foam were carefully analyzed. Radiation-cross-linked polypropylene is shown to be suitable for *sc*CO_2_ foaming by increasing the melt strength and decreasing the crystallinity of polypropylene.

## 2. Results and Discussion

### 2.1. Gel Content and Melt Flow Index Analyses

**Figure 1:**
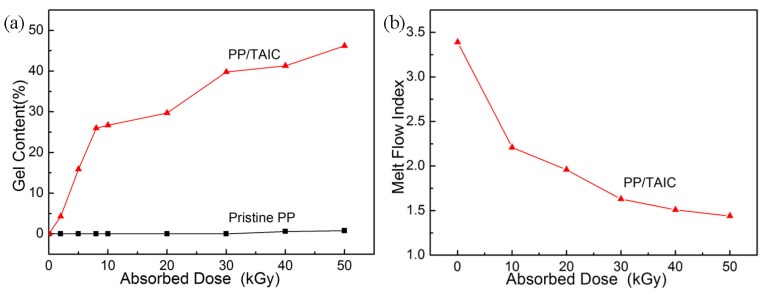
Effect of absorbed dose on the pristine PP and PP/TAIC (the amount of TAIC was 2 wt %) blends: (**a**) gel content and (**b**) melt flow index.

The effect of the amount of absorbed dose on the gel content of PP is shown in Figure 1a. The gel content of PP/TAIC (the amount of TAIC was 2 wt %) increases gradually through doses up to 50 kGy. A higher gel content corresponds to a higher portion of the network formation in the amorphous region of polypropylene, which was insoluble in solvents. Because the crystalline portion of polypropylene was soluble in boiling xylene, we did not obtain any gel content for non-cross-linked PP. Macromolecules in the crystalline regions moved into adjacent amorphous regions [16], and the macromolecules intertwined together and formed a 3D network structure in the amorphous regions, as can also be seen from the change of crystallinity listed in Table 1.

The melt flow indices (MFI) of radiation cross-linked PP samples at 0–50 kGy are shown in Figure 1b. The MFI value decreases with the amount of absorbed dose, indicating clear cross-linking of PP by irradiation. However, the MFI of pristine PP after irradiation in air could not be measured because of significant radiation degradation and oxidation. Unlike polyethylene, PP easily undergoes degradation upon irradiation in air. The addition of a cross-linking agent, such as TAIC, possessing multi-functional groups is required to obtain an insoluble gel of PP.

### 2.2. Melting Point and Crystallinity

**Figure 2:**
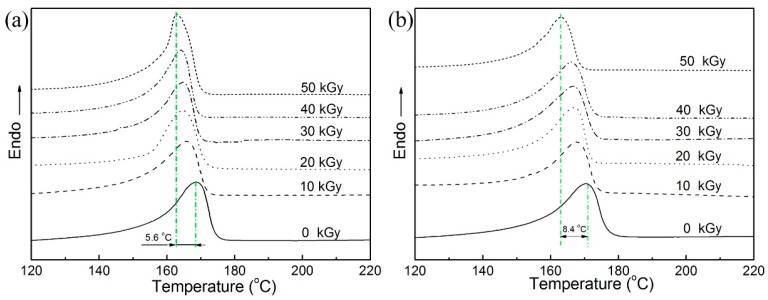
Differential scanning calorimetry (DSC) curves of the melting transition of PP samples at different doses: (**a**) pristine PP and (**b**) PP/TAIC (2 wt %).

Differential scanning calorimetry (DSC) curves of the melting transitions of PP samples at 0–50 kGy are shown in Figure 2. The decrease in the melting point of pristine PP after irradiation is ascribed to the radiation degradation of PP in the crystalline regions, as indicated in Figure 2a. The decrease of the MFI of PP/TAIC is due to the moving of macromolecular chains from crystalline to amorphous regions, which destroyed the crystal structure and are independent of crystallinity [16]. The crystalline region might also have some less-perfect regions, which decreased the melting points [24]. The decrease in the melting point of cross-linked PP is larger than that of the pristine PP, and is attributed to the lower crystallinity of cross-linked PP.

The degrees of crystallinity (X_c_) of irradiated PP samples are listed in Table 1. The crystallinity of irradiated PP/TAIC decreased with increasing doses, because of the degradation of macromolecular chains in the crystalline region. Frequency of motion and rearrangement of macromolecular chains were strong in PP, and the macromolecular chains moving from the crystalline regions folded and wound to form cross-linking segments in the amorphous regions [16].

### 2.3. Dynamic Shear Rheological Properties

**Figure 3:**
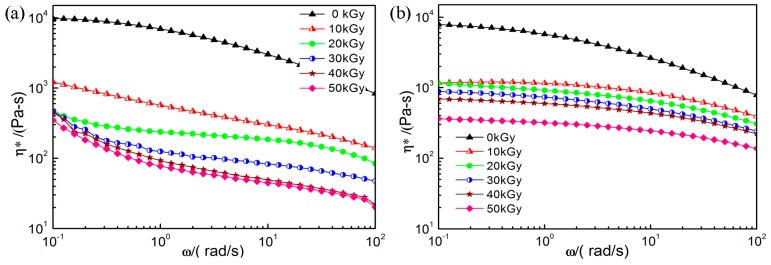
Amplitude oscillatory shear rheological properties at different doses: (**a**) pristine PP and (**b**) PP/TAIC (2 wt %).

Dynamic shear rheological properties are crucial to determine the optimal conditions to fabricate well-defined PP foam materials. Figure 3 shows the small amplitude oscillatory shear rheological properties of pristine PP and PP/TAIC at 0–50 kGy. The figure shows the complex viscosity as a function of frequency ranging from 10^−1^ to 10^2^ rad/s. It can be seen that the zero shear viscosity of PP is higher than that of PP/TAIC, due to the presence of the cross-linking agent. The shear viscosity decreased nearly by an order of magnitude at 10 kGy. The reduction in viscosity becomes less significant as the dose is further increased from 10 to 50 kGy, due to the cross-linking in the amorphous regions. It is also seen that the complex viscosity of PP/TAIC is higher than that of pristine PP. Generally, the addition of TAIC can increase the complex viscosity of PP after irradiation.

### 2.4. Morphology of Cross-Linked PP Foams

**Figure 4:**
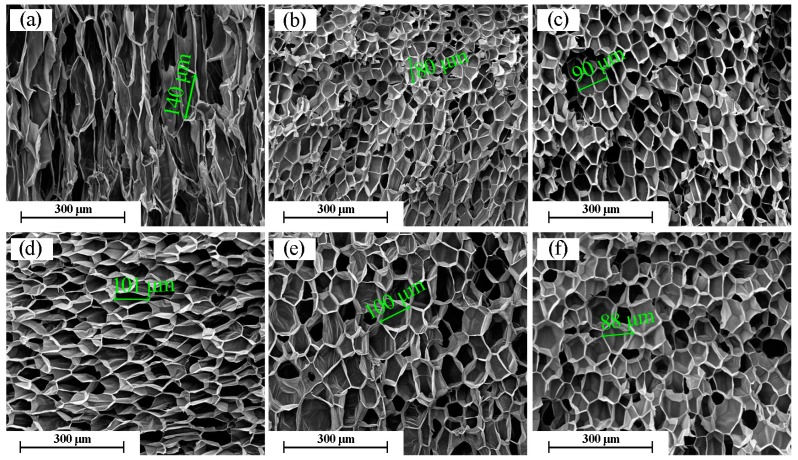
SEM micrographs for PP/TAIC (2 wt %) blend foams produced at 152 °C and 20 MPa and at different doses (kGy): (**a**) 0; (**b**) 10; (**c**) 20; (**d**) 30; (**e**) 40; and (**f**) 50.

Cell morphologies of the foamed PP samples were characterized using scanning electron microscopy (SEM). The effect of absorbed dose on the cell morphology of PP foam is shown in Figure 4. The cell structure was greatly improved, and the cells were more uniform as the absorbed dose increased from 0 to 30 kGy. The cells of the foamed PP materials became more uniform, and more closed cells were present in the foamed PP compared to the cell structure of the foamed blends of PP/nanoclay and PP/nanocrystalline cellulose by supercritical carbon dioxide-foamed PP [21,22]. Moreover, the cells have a relatively stable diameter for doses from 10 kGy to 40 kGy, owing to a larger cross-linked region and higher melt strength of PP, which is beneficial for bearing the pressure of CO_2_. Therefore, more closed cells are formed with thick cell walls after the release of the pressure.

**Figure 5:**
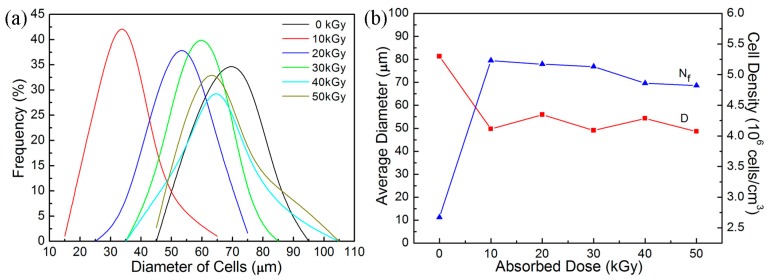
Effect of absorbed dose (**a**) on the cell size distribution and (**b**) on the cell diameter and cell density of the PP/TAIC foams produced at the same conditions in Figure 4.

Figure 5a shows the effects of absorbed dose on the cell size distribution of PP/TAIC foamed at 152 °C and 20 MPa. The cell size distribution is dependent on the dose. The width of the cell size distribution (which indicates the uniformity of cell size) shows that the widest cell size distribution of the non-cross-linked PP and the width decrease slightly with the dose. These results indicate a better uniformity of cells for cross-linked PP foam. It is also shown that the cell size of cross-linked PP foam is smaller than that of the non-cross-linked PP foam. The average cell diameter of cross-linked PP foam undergoes a slight change as the dose increases from 10 to 50 kGy, as shown in Figure 5b. The higher melt strength of cross-linked PP restrains the cell size expansion of PP foam, causing the average diameter of cross-linked PP foam to be smaller than that of non-cross-linked PP [9].

### 2.5. Morphology of Pristine PP Foams

**Figure 6:**
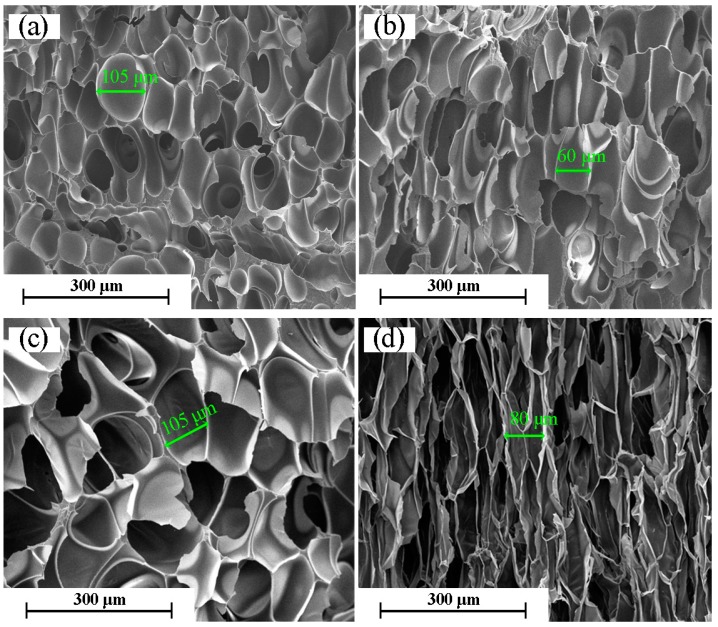
SEM micrographs for the foaming of PP (without TAIC) irradiated at different doses (kGy): (**a**) 10; (**b**) 20; (**c**) 30; and (**d**) 40.

The SEM images in Figure 6 show the cell morphology of non-cross-linked PP foams at 10–40 kGy. For the non-cross-linked PP foams, we observe that the cell structure of the foam is non-uniform, and the cells severely collapse. Additionally, the cell size is larger compared to the cross-linked PP foams shown in Figure 4. This can be explained by radiation-induced degradation and the reduced melt strength of PP. The low melt strength results in cell rupture and cell merger in PP foam.

### 2.6. Volume Expansion Ratio and Foaming Rate of PP Foam

**Figure 7:**
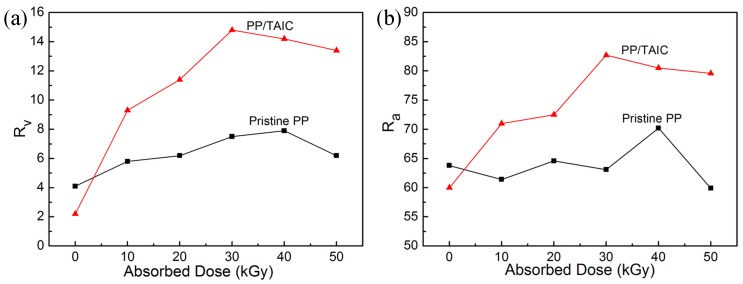
Dose dependence of (**a**) volume expansion ratio (R_v_) and (**b**) foaming rate (R_a_) for pristine PP and PP/TAIC (2 wt %) foams prepared at 152 °C and 20 MPa.

The volume expansion ratio of foamed PP is shown in Figure 7a. The volume expansion ratio (R_v_) of foamed PP/TAIC increases with doses up to 30 kGy. Figure 7b shows the dose dependence of foaming rates for different formulations. PP/TAIC has a higher foaming rate, and the optimum dose for the foaming of PP/TAIC is 30 kGy. With the addition of TAIC, the radiation induces cross-linking and branching of PP [15,25], which, in turn, enhances the chain entanglement and interaction among macromolecules and improves the melt strength and viscosity [24]. The volume expansion ratio and foaming rate of the cross-linked PP show a slight decrease at 40 kGy and 50 kGy. This decrease of the volume expansion ratio is ascribed to the higher gel content or degree of cross-linking at high doses, and the cross-linking segments in PP may become rigid, which is bad for the foaming of PP. Moreover, the intertwined macromolecular chains of PP may hinder the nucleation and the expansion of the cells as the CO_2_ runs out of the melt PP. The gel content was 39.8% at 30 kGy, which may be close to the optimum gel content of PP for *sc*CO_2_ foaming. We suggest that a suitable gel content of PP for *sc*CO_2_ foaming is 25%–45%.

### 2.7. Foaming Temperature Window of the Cross-Linked PP

**Figure 8:**
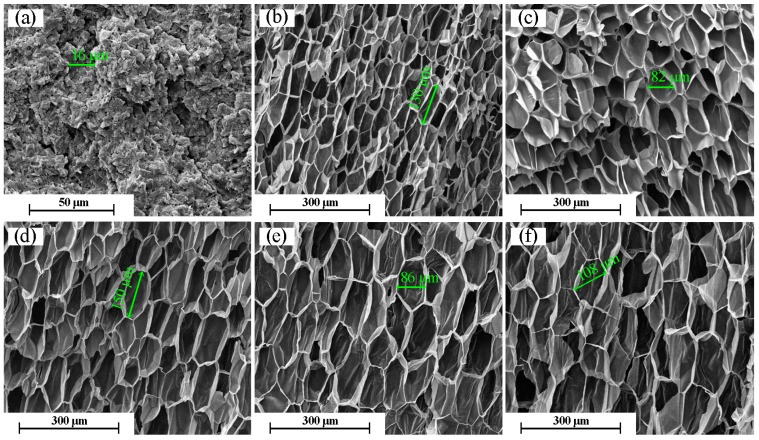
Effect of the foaming temperature on the cell morphology of the PP/TAIC (2 wt %) at 20 MPa (30 kGy). Foaming temperature: (**a**) 146 °C; (**b**) 148 °C; (**c**) 150 °C; (**d**) 152 °C; (**e**) 154 °C; and (**f**) 156 °C.

The foaming of PP/TAIC irradiated at 30 kGy was further investigated at different temperatures in order to better understand the foaming behavior of cross-linked PP. Figure 8 shows a comparison of cell structures obtained at different foaming temperatures and a saturation pressure of 20 MPa. It is seen that PP/TAIC cannot be foamed at 146 °C, but can be well-foamed at 148–154 °C. At 156 °C, the cell structure is relatively intact, but some cracks are observed on the cell walls. The results imply that the foaming temperature window of radiation cross-linked PP increases to 8 °C, compared to the 4 °C for non-cross-linked PP [4]. The increase of the foaming temperature window is due to the higher melt strength and decreased crystallinity of cross-linked PP.

### 2.8. Comparison of Pristine PP Foam and Cross-Linked PP Foam

**Figure 9:**
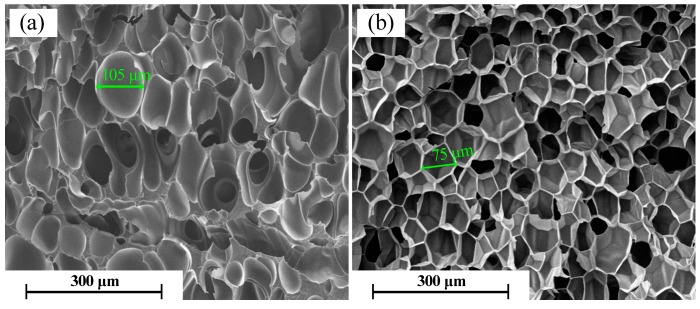
SEM micrographs for two foaming specimens of foamed PP, prepared at 152 °C and 20 MPa: (**a**) non-cross-linked and (**b**) cross-linked (30 kGy).

Figure 9 shows micrographs of the fracture surfaces of non-cross-linked and cross-linked (30 kGy) PP foam prepared at 152 °C and 20 MPa. For the non-cross-linked PP foam, the cell fusion and breaking-up of the cell walls can be clearly seen, as the melt strength of PP is too low to bear the pressure release of CO_2_ at 152 °C. In the case of cross-linked PP foam, the cells continue to be intact polygonal closed-cell structures, due to the high melt strength of PP. This is the reason that the cross-linked PP can be foamed over a relatively wider temperature window.

## 3. Experimental Section

### 3.1. Materials

Polypropylene (pellet, isotactic, homopolymer) T30s, with a density of 0.91 g/cm^3^ and a melt flow index of 3.0 g/10 min at 230 °C with a weight of 2.16 kg [16], was purchased from Sinopec Shanghai Chemical Co. (Shanghai, China). Triallylisocyanurate (TAIC, C_12_H_15_N_3_O_3_), with an average molecular weight of 249.27 g/mol and a density of 1.11 g/cm^3^, was purchased from Shanghai Farida Chemical Co., Ltd. (Shanghai, China). Carbon dioxide with a purity of 99.5% was supplied by Xiang Kun Special Gases of Shanghai (Shanghai, China). Xylene, with a purity of 99.6%, was purchased from Sinopharm Chemical Reagent Co., Ltd. (Shanghai, China).

### 3.2. Sample Preparation

PP pellets were first mixed with the cross-linking agent (PP/TAIC weight ratio: 49/1) at 180 °C using a mixer (Thermo Haake PolyDrive, Tianjin, China). The mixture was hot pressed into a 2 mm thick sheet at 195 °C and 10 MPa for 15 min and cooled to ambient temperature. A series of as-obtained PP sheets were irradiated with Co-60 γ-rays (Shanghai Institute of Applied Physics) under a nitrogen atmosphere at room temperature. The dose rate was 0.114 kGy/min.

### 3.3. Gel Content Measurement

The gel content of polypropylene was measured according to ASTM D 2765-95. Xylene was used as an extraction solvent, and the extraction was performed at the boiling point of xylene (140 °C) for 72 h [26,27]. After extraction, the solid remainder was dried to a constant weight at 60 °C and then reweighed. The gel content was calculated as follows:

(1) $$\mathrm{Gel}\;\mathrm{content}\;\left(\%\right)=\frac{M_{1}}{M_{0}}\;\times 100\%$$

where $M_{0}$ is the initial weight of PP, and $M_{1}$ is the weight of the insoluble part.

### 3.4. Melt Flow Index (MFI)

A plastometer (Zwick 4100, Shenzhen, China) apparatus was used to test the MFI of the original and irradiated PP samples at 230 °C under 2.16 kg of weight, according to ASTM D-1238 [28].

### 3.5. Melting Point and Crystallization

A NETZSCH STA 449 F3 Jupiter (Shanghai, China) differential scanning calorimeter (DSC) equipped with a data station was used to scan the melting transitions of γ-irradiated samples (10–15 mg) in aluminum pans. The samples were heated from 25 to 200 °C at a heating rate of 10 °C/min under an argon flow (20 mL/min). The crystallinity was calculated as follows:

(2) $$X_{c}\left(\%\right)=\frac{\Delta H_{f}}{\Delta H_{f0}}\times 100\;\%$$

where $\Delta H_{f}$ is the melting enthalpy measured in the heating experiments, and $\Delta H_{f0}$ is the theoretical value of enthalpy of 100% crystalline polypropylene, which has a value of 207.1 J/g [29].

### 3.6. Rheological Characterization

A rotational rheometer (ARES-G2, TA Instruments-Waters LLC, Shanghai, China) with a plate-plate geometry of 20 mm in diameter and a gap of 1 mm, under nitrogen protection at 180 °C, was used to determine the dynamic rheological properties. All the tests of the linear viscoelastic region of the PP materials were performed at stresses. Amplitude oscillatory shear tests were performed over a frequency range of 100–0.1 rad/s [30].

### 3.7. Foaming Process

**Figure 10:**
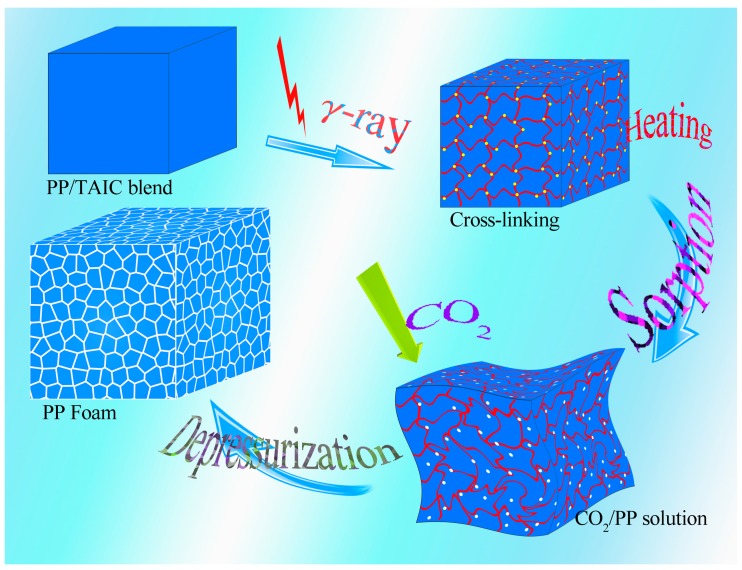
Preparation of microcellular PP foam using the supercritical carbon dioxide (*sc*CO_2_) method.

The foaming of PP sheets was conducted in an autoclave by using supercritical CO_2_ as the blowing agent. First, the high-pressure *sc*CO_2_ was pumped into the autoclave and kept at 20 MPa. The temperature of the PP sample (thickness: 2 mm) was heated to a pre-set temperature at a heating rate of 10 °C/min, and the sorption time was 90 min; under these conditions, CO_2_ saturation should reach equilibrium [31,32]. Then, the depressurization rate was controlled by a solenoid valve, and the value was 10 MPa/s in this work. The supercritical experimental apparatus was schematically depicted in previous papers [33,34]. The complete foaming process of PP in this work consists of the following steps: (1) plasticizing, (2) sheet molding, (3) irradiation, (4) foaming, and (5) stable microporous structure formation. The preparation of PP foam in this work is shown in Figure 10.

### 3.8. Morphology of Foam

Scanning electron microscopy (SEM; Zeiss MERLIN Compact 14184, Carl Zeiss Jena, Shanghai, China) was used to observe the cell morphologies of the foamed PP samples. Foaming samples were immersed in liquid nitrogen for 2 min, fractured at liquid nitrogen temperature, and then mounted on stubs. Fractured surfaces were sputter-coated with a 10-nm layer of gold. Foams with uniform cell morphologies were also characterized in terms of cell density and average cell size. Image Pro-plus was used to analyze the SEM images. The average cell diameter, D, in each micrograph was calculated using the following equation:

(3) $$D=\frac{\sum d_{i}n_{i}}{\sum n_{i}}$$

where $n_{i}$ is the number of cells within a perimeter-equivalent diameter of $d_{i}$.

The cell density ($N_{f}$) was determined by the number of cells per unit volume of foam, which was calculated as follows:

(4) $$N_{f}={\left(\frac{nM^{2}}{A}\right)}^{3/2}$$

where n, M, and A are the number of cells in the micrograph, magnification of the micrograph, and area of the micrograph (cm^2^), respectively [35]. The foaming rate of the foamed PP, $R_{a}$, was the ratio of the sum of the areas of bubble holes to the total area, and was calculated as follows:

(5) $$R_{a}=\;\frac{\sum_{i}A_{N_{i}}}{A}$$

where $A_{N_{i}}$ and A are the sum of the area of the bubble holes and the area of the image in the field of vision, respectively [34].

The volume expansion ratio of the foamed PP, $R_{v}$, was the ratio of the bulk density of pristine PP ($\rho_{s}$) to that of the foamed PP ($\rho_{f}$), calculated as follows:

(6) $$R_{v}=\frac{\rho_{s}}{\rho_{f}}$$

The mass density of pristine PP ($\rho_{s}$) and foamed PP samples ($\rho_{f}$) were determined by deducing the Archimedes Law, involving weighting polymer foam in water with a sinker by using an electronic analytical balance (HANG-PING FA2104, Shanghai, China).

## 4. Conclusions

The foaming behavior of radiation cross-linked isotactic polypropylene with the addition of a cross-linking agent (TAIC) by the *sc*CO_2_ foaming method was studied. Radiation cross-linking of PP was shown to result in an obvious change in the gel content and crystallinity. These variations had a positive effect on the cell structure—including cell size distribution, cell size, and cell density—and the foaming rate for *sc*CO_2_ foaming of PP. In particular, radiation cross-linking led to a wider foaming temperature range (8 °C) of isotactic PP compared to the pristine PP (4 °C). The results of this work are useful for the design of *sc*CO_2_ foaming parameters of polypropylene.

## Figures and Tables

**Table 1:** Melting point (T_m_) and degree of crystallinity (X_c_) of pristine polypropylene (PP) and PP with the addition of triallylisocyanurate (PP/TAIC) irradiated at different doses.

Dose (kGy)	Pristine PP		PP/TAIC	
	X_c_ (%)	T_m_ (°C)	X_c_ (%)	T_m_ (°C)
0	58.1	169.1	61.8	171.0
10	49.7	166.7	41.5	168.4
20	43.5	164.9	41.2	167.5
30	42.6	164.9	34.9	166.7
40	46.6	164.2	32.4	165.1
50	41.8	163.5	33.7	164.0
